# Native Yeasts and Lactic Acid Bacteria Isolated from Spontaneous Fermentation of Seven Grape Cultivars from the Maule Region (Chile)

**DOI:** 10.3390/foods10081737

**Published:** 2021-07-28

**Authors:** Wendy Franco, Sergio Benavides, Pedro Valencia, Cristian Ramírez, Alejandra Urtubia

**Affiliations:** 1 Departamento de Ingeniería Química y Bioprocesos, Pontificia Universidad Católica de Chile, Ave. Vicuña Mackena 4860, Santiago 7820244, Chile; 2 Departamento de Ciencias de la Salud, Carrera de Nutrición y Dietética, Pontificia Universidad Católica de Chile, Ave. Vicuña Mackena 4860, Santiago 7820244, Chile; 3 Núcleo de Investigación en Agroalimentos y Nutrición Aplicada, Universidad Adventista de Chile, Camino a las Mariposas km 12, Chillán 3780000, Chile; sergiobenavides@unach.cl; 4 Departamento de Ingeniería Química y Ambiental, Universidad Técnica Federico Santa María, Ave. España 1680, Valparaíso 2390123, Chile; pedro.valencia@usm.cl (P.V.); cristian.ramirez@usm.cl (C.R.); alejandra.urtubia@usm.cl (A.U.)

**Keywords:** native yeasts, Chile, wine, non-*Saccharomyces*, lactic acid bacteria, *Candida oleophila*

## Abstract

Grapes are a source of native yeasts and lactic acid bacteria (LAB); however, the microbial make up is dependent on the grape cultivar and the regional growth conditions. Therefore, the aim of this study was to characterize the yeast and LAB in seven grape cultivars cultivated in Chile. Grape juices were fermented at 25 °C for 7 days. Samples were collected to analyze sugar, organic acids, and ethanol. Microbial evolution was measured with culture-dependent and molecular approaches. Then, a native isolated *Candida oleophila* was selected for further sequential fermentations with *Saccharomyces cerevisiae*. The grape cultivars in the Maule showed a diversity of non-*Saccharomyces* yeasts, with a greater diversity observed at the beginning of the fermentation. However, species from the *Hansenasporia*, *Metschnikowia*, *Torulaspora*, *Lachancea*, and *Candida* genera were detected after 7 days, suggesting tolerance to environments rich in ethanol, capability may be associated to the terroir studied, which is characterized by torrid weather and antique and traditional vineyards. The alcoholic fermentation negatively impacted the LAB population, and after 7 days only *Leuconostoc mesenteroides* was isolated. In the sequential fermentations, *C. oleophila* was able to produce fermented grape juices with <1.5 g/L glucose, 12.5% (*v*/*v*) alcohol, and low concentrations of malic (<1.00 g/L) and succinic (2.05 g/L) acids, while acetic acid reached values >0.3 (g/L). To our knowledge this is the first time *C. oleophila* has been reported as a potential starter culture for wine production. However, more studies are necessary to fully characterize the potential of *C. oleophila* on wine attributes.

## 1. Introduction

Wine fermentation is a process that develops from a complex mixture of native microorganisms, which act sequentially and proliferate or decline as fermentation progresses. The establishment of yeasts and LAB during the fermentation process determines the wine quality and specific attributes [1]. In order to better control the fermentation, commercial strains (usually *Saccharomyces* spp.) are used. However, in recent years, several non-traditional strains have been studied and applied for winemaking, with the aim to enrich wine aromatic and flavors profiles [2,3].

Non-*Saccharomyces* yeasts are often autochthonous and can be found and isolated from grapes, spontaneous fermentations, and winery equipment [4,5,6]. Among these, the greatest diversity of yeasts can be found on grapes and fresh grape juice. Diversity is associated with climate conditions (rainfall, temperature) [7], *terroir* [8,9], maturity of the grapes [10], damage caused by insects or birds [11], and chemical agents that can be used during the grapes’ cultivar [11,12]. Distinct yeast profiles might be related to the unique conditions according to the geographical and agricultural crop procedures used in each vineyard.

Although these native yeasts are less competitive than *S. cerevisiae*, they play a significant role during the early fermentation stages. They can influence the characteristics of the resulting wine by producing extracellular enzymes and metabolites of oenological significance, and therefore modify sensory and organoleptic properties [4,13,14,15,16,17,18]. The wine industry is actively seeking for diversification of its wine offer, targeting premium wines with specific flavor profiles. In that sense the use of non-*Saccharomyces* yeasts is of interest [19]. These yeast are able to convert non-volatile compounds into volatile aroma, therefore influencing the varietal flavors [20,21,22].

Even though selection and isolation of non-*Saccharomyces* yeasts is commonly done from grapes and fresh grape juice, in recent years, the characterization of spontaneous fermentations has led to the isolation a greater number of non-*Saccharomyces* yeast species [10,14,23,24,25,26,27,28]. The isolation of yeasts from spontaneous fermentations permits the identification of strains that show desirable attributes for winemaking during alcoholic fermentation [13,22,29,30]. However, in order to use non-*Saccharomyces* strains, some attributes need to be accomplished. The yeasts need to be competitive enough to grow along with *S. cerevisiae* and other microbiota present in the grape juices, to tolerate certain levels of alcohol to avoid stuck fermentations, and to grow at high sugar concentrations. They also resist sulfur dioxide that is commonly added to fresh grape juices as part of the standardization procedures, produce low quantities of hydrogen sulfide, and result in wines with low volatile acidity, among others [15].

About 40 non-*Saccharomyces* yeasts isolated from different sources have been studied [2]. The most common genera isolated and characterized are *Hanseniaspora* [16,31,32,33,34], *Torulaspora* [13,17,18,35,36], *Metschinokowia* [37,38,39,40], and *Lachancea* [13,41,42,43]. These yeasts have been used in single, co-culture, and sequential fermentations, and the findings led to a better understanding of their role in winemaking and the positive and/or negatives effect they might exert [2,44,45,46,47].

On the other hand, lactic acid bacteria (LAB) play an important role during and after the alcoholic fermentation, by producing lactic acid from the decarboxylation of the malic acid left in the fermented grape juice and thus increasing the wine pH. Although species of *Oenococcus* are the most fitted to conduct the malolactic fermentation (MLF), other LAB genera are also involved in the process (*Lactobacillus*, *Leuconostoc*, *Pediococcus,* and *Weissella*). During alcoholic fermentation, the LAB evolution is influenced by the yeast diversity, and bacterial–yeast interactions might affect the malolactic fermentation during this stage [48].

Ecological surveys have been carried out extensively with the aim of characterizing the microbiota from grapes, grape juices, and wines from different regions [10,11,25,26,27,28,48,49,50]. However, to our knowledge, little information is available for wines produced in South America, and in particular from Chile. Jara et al., (2016) studied the presence of five non-*Saccharomyces* genera (*Metschnikowia*, *Hanseniaspora*, *Torulaspora*, *Debaryomyces*, *Meyerozyma*, and *Rhodotorula*) in grape juice from different regions in Chile [51]. The authors found that all the genera were present, but with different abundances, probably associated with the climate conditions surrounding the vineyards. Although the importance of the study, no information associated to what happens to the yeasts diversity during the alcoholic fermentation has been reported. Similarly, for bacteria, few studies have reported if and how the alcoholic fermentation influence the bacterial diversity [51].

In Chile, wine is the second largest food export commodity, and the country’s production is rich and varied. Wine elaboration is mostly carried out using commercial *S. cerevisiae* strains with few commercial non-*Saccharomyces* yeasts used. Given the microbial diversity and the potential of native non-*Saccharomyces* yeasts over wine attributes, the goals of this study were (1) to isolate native yeasts and LAB present in different Chilean grapes commonly used for wine production, focusing on the microbial composition at the beginning and during spontaneous alcoholic fermentation; (2) to characterize the alcoholic fermentative profile of selected yeast isolates in laboratory fermentations; and (3) to study (at a laboratory scale) the ability of selected isolates to ferment grape juice in sequential fermentations with *S. cerevisiae*. The long-term goal of these initial studies is to characterize native microorganisms with potential as cultures for the elaboration of wines with distinct attributes.

## 2. Materials and Methods

### 2.1. Grape Berries Samples and Grape Juice Preparation

**Figure 1:**
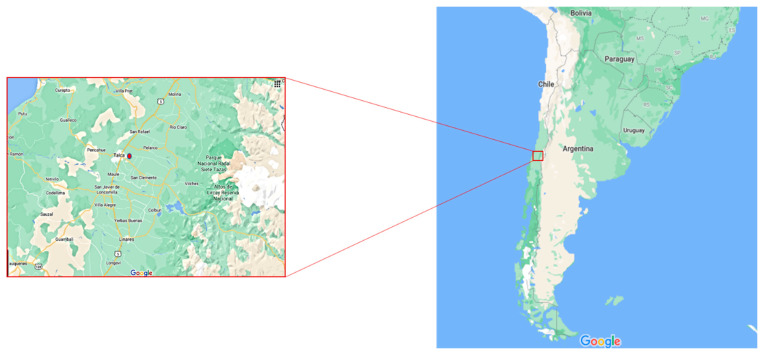
Location of the vineyard samples from which grapes were collected.

Grape berries were collected from the Palo Alto Winery located in the Maule Region (35°26′ S 71°40′ W, Chile, Figure 1) during the 2013 vintage season (March to May). Seven grape varieties were chosen (Cabernet Sauvignon, Carignan, Chardonnay, Merlot, Pinot, Syrah, and S. Blanc). Sampling for each variety was conducted as follows. Within a barrack, four equidistant streets were selected. On each street, 10 grape clusters were randomly hand-picked following a zig-zag pattern. Samples were collected in triplicate and transported refrigerated to the Food Microbiology Laboratory at Pontificia Universidad Católica de Chile (Santiago, Chile). Upon arrival, clusters’ stems and leaves were separated manually from each grape. The berries were processed using a food blender MiniPymer (Oster, China) at minimal speed. Extra care was taken to not break seeds. The resulting pulp was then filtered with a gauze to separate seeds and large particulates. The grape juice’s initial nitrogen content was adjusted to 250 mg/L of yeast amino nitrogen (YAN) with diammonium phosphate salts (Sigma Aldrich, Saint Louis, MO, USA) and the pH was adjusted to 3.5.

### 2.2. Spontaneous Fermentation

In triplicate, 400 mL of grape juice was placed in a sterile 500 mL Erlenmeyer flask provided with an airlock. Flasks were incubated in an orbital shaker incubator (Stuart, Staffordshire, UK) at 100 rpm at 25 °C for 7 days. Aliquot samples were collected daily.

#### 2.2.1. Chemical Analysis

The pH of the samples was monitored using a pH meter (Accumet Fisher Scientific, Pittsburgh, PA, USA). Organic acids, sugar, ethanol, and glycerol concentrations were measured with high-performance liquid chromatography (HPLC) using an Amimex HPX/87H anion-exchange column (Bio/Rad Laboratories, Hercules, CA, USA). The column temperature was held at 55 °C, and sample components were eluted with 5 mM sulfuric acid at a flow rate of 0.5 mL/min. A LaChrom L-7450A diode array detector (Hitachi, Tokyo, Japan) set at 210 nm was used to quantify organic acids. A LaChrom L-7490 refraction index detector (Hitachi) connected in series with the diode array detector was used to measure glucose, fructose, ethanol, and glycerol. External standardization of the detectors was done using six concentrations of the standard compounds [52].

#### 2.2.2. Culture-Dependent Analysis

##### Microbiological Characterization

For the microbial characterization, the method reported by Franco et al., (2012) was followed. Briefly, collected samples were serially diluted in 0.01% peptone water (Sigma Aldrich) and spread platted. Yeasts were enumerated using yeast extract glucose chloramphenicol agar (YGC agar, Sigma Aldrich) and Wallerstein Laboratory nutrient agar (WLN agar, Oxoid, Hampshire, UK) supplemented with diphenyl (500 mg/mL) to prevent mold growth [53]. Plates were incubated aerobically at 25 °C for at least 48 h or until colonies were observed on plates. LAB were enumerated using de Man, Rogosa, and Sharpe agar (MRS agar, Oxoid) supplemented with 1% cycloheximide (0.1% solution, Oxoid) to inhibit yeast growth. MRS agar plates were incubated anaerobically using gas packs (Oxoid) at 30 °C for 48 h or until colonies were observed [54].

##### Isolation and Identification of Microorganisms

The colonies visually observed in the agar plates were grouped according to their morphology. Four characteristic parameters were used for the classification: color, shape, elevation, and margin [42]. Four to five independent clones (colonies) of the representative morphology types were picked and streaked in the respective culture medium. Frozen stocks of all isolates were prepared in MRS and YGC broth for BAL and yeasts, respectively, containing 15% glycerol (Sigma Aldrich).

Microbiological isolates (yeasts and bacteria) were identified through partial sequencing of the 26S or 16S rDNA, respectively. Chromosomal DNA was obtained using GeneJET Genomic DNA Extraction and Purification Kit (Thermo Scientific, #K0722, Hampshire, UK). Both bacterial and yeast DNA were amplified by polymerase chain reaction (PCR) under the following conditions. For the bacterial isolates, the forward and revers primers (27F/1492r for the bacterial isolates [55,56]) were mixed with the chromosomal DNA. The PCR conditions were stage 1: 94 °C for 3 min; stage 2: 94 °C for 1 min, 57 °C for 2 min, 72 °C for 2 min, repeated 25 times; stage 3: 72 °C for 7 min; and stage 4: 4 °C until use. For the yeast isolates, the forward and reverse primers NL-1/NL-4 were used [57] under the following PCR conditions: stage 1: 95 °C for 5 min; stage 2: 95 °C for 1 min, 52 °C for 45 s, 72 °C for 1 min, and repeated 35 times; stage 3: 72 °C for 7 min; and stage 4: 40 °C until use. All primers were obtained in Macrogen (Seoul, Korea). The PCR products were purified using the Qiagen PCR purification kit and sequenced by Macrogen (Seoul, Korea). The sequences obtained were subjected to the basic local alignment search tool (BLAST 2.2.26) [58] in the GenBank [59] non-redundant nucleotide database for yeast cultures and the 16S rRNA microbial database for bacterial cultures to determine identities. Only alignment matches with 95% identity or higher, with no gaps, and expected values of 0.0, were considered for identification purposes. The sequences are available at the National Center for Biotechnological Information (NCBI) GenBank‘ through their access numbers.

#### 2.2.3. Culture-Independent Analysis

##### Preparation and Sequencing of the Grape Juices’ Fermentation rDNA Libraries

Samples collected at the beginning of the fermentation (day 0), and after 7 days, were subjected to DNA extractions and sequencing data processing. The protocol reported by Franco et al., (2020) was followed for the preparation and sequencing of the grape juice’s fermentation rDNA libraries [60]. Briefly, cells harvested from 10 mL of fermented grape juice were re-suspended in sterile saline and were treated to eliminate dead bacterial/yeast and extracellular DNA with 2.5 mM propidium monoazide (PMA) stock solution (1.3 mg/mL PMA in 20% DMSO; Biotium, Inc., Hayword, CA, USA) following the method described by Pan and Breid (2001) [61]. PMA-treated samples were stored as cell pellets at −20 °C until DNA extraction was performed. Total genomic DNA was extracted using a MasterPure^TM^ DNA Purification Kit (Epicentre, Madison, WI, USA). The 16S rDNA gene regions V3 to V4 were amplified by PCR using the Bakt_341F/Bakt_805R primers [62]. Primers were barcoded as described by the manufacturer for the Illumina MiSeq sequencing technology. For the fungi analysis, primers ITS1F/ITS2 were used. Overall, 181 samples, corresponding to the fermentation of 7 grapes cultivars, including Cabernet Sauvignon, Carignan, Chardonnay, Merlot, Pinot, Syrah, and S. Blanc, were processed. Samples collected at the beginning of the fermentation (day 0) and after 7 days were subjected to extraction and sequencing of DNA.

##### Processing of the 16S rDNA Amplicon Sequences Data Corresponding to Spontaneous Fermentation Samples

Processing of the 16S rDNA amplicon sequences was conducted with the methodology followed by Franco et al., (2020) [60]. Briefly, the reads from fermentation samples were quality trimmed with Trimmomatic (version 0.36), and any reads with lengths less than 36 bp were removed [63]. Primer sequences were removed using MacQIIME 1.9.1-20150604 [64]. The reads were merged using fastq-join with the default parameters [65], and merged reads with Phred quality scores less than 20 were removed. VSearch [66] was used to de-replicate the sequences and remove singletons, sort them by sequence abundance, and cluster the sequences at 97%. The most abundant read from each cluster was selected as the cluster centroid and, with VSearch, chimeras were removed from the list of centroids. The remaining sequences served as a set of representative sequences. The read sequences were mapped to the representative sequences to generate the orthologue taxonomic units (OTUs) data. Taxonomy was assigned to the OTUs using the SILVA (version 128) database [67,68]. The OTUs that represented less than 0.005% of the total sequences were removed, along with those that corresponded to chloroplasts and/or mitochondria. MacQIIME 1.9.1-20150604 was used to calculate the alpha diversity metrics, including phylogenetic diversity (PD), Chao1, and the observed OTUs count to assess the sampling depth. PYNAST was used to align the sequences using the SILVA (version 128) core alignment sequences [69]. Based on the resulting alpha diversity plots, a rarefaction level of 20,000 sequences per sample was selected.

##### ITS Amplicon Sequences Data Processing, Corresponding to Grape Juice Fermentation Samples

Regarding the fungal analysis, the readings that were below 36 bp were discarded in the analysis. Regarding the forward and backward reads, they were fused with an overlap of 50 bp, eliminating the quality reads less than 20 and 200 bases in length. For reading clipping, an ITS Extractor (ITSx; Bengtsson-Palme et al., 2013) was used. All the results obtained were processed as described for the bacterial analysis. However, the UNITE database (https://unite.ut.ee/repository.php accessed on 20 November 2016) was used for the taxonomic assignment. For this analysis, OTUs smaller than 0.005% of the total sequences were discarded. Samples were rarefied to 7000 reads for Beta and Alpha diversity analysis for samples containing more than 1000 reads.

### 2.3. Alcohol Production by the Selected Non-*Saccharomyces* Isolates

The defined medium was fermented with selected isolates (75 g/L glucose, 75 g/L fructose, 3 g/L tartaric acid, and 6.76 g/L yeast nitrogen base, at a pH of 3.5) [70]. Strains were screened in single cultures in a 250 mL sterile fermentation flask provided with airlocks. The inoculum was prepared with overnight yeast cultures (18–24 h at 25 °C), centrifuged, and washed twice with peptone water (0.01%). The resulting pellets were re-suspended in the same solution. The inoculate were transferred to the defined medium to 5 log CFU/mL and incubated at 25 °C for 4 days. Samples were collected and analyzed for pH and sugar and ethanol concentrations as described in Section 2.2.3. Inoculations with a commercial *S. cerevisiae* (Actiflore BO213, Laffort, Bourdeux, France) were used as the control.

### 2.4. Non-Saccharomyces Isolates Sequential Fermentations with S. cerevisiae in Carmenere Grape Juice

Two selected non-*Saccharomyces* yeasts belonging to the *Candida* genera isolate were evaluated in sequential fermentation with the commercial *S. cerevisiae* yeast in Carménère grape juice (Santa Emiliana Winery, Chile). This grape juice was selected because is the signature wine of Chile [71] and, therefore, there is an industrial interest to contribute with unique attributes to the wine variety that might be given by non-*Saccharomyces* yeasts. The grape juice’s YAN was adjusted to 250 mg/L and 35 mg/L of free SO_2_ was added using a sodium metabisulphite solution (2%, Sigma Aldrich), and the pH was adjusted to 3.55. Sequential fermentations were carried out in 4 L bioreactors (Applikon Biotechnology, Dover, NJ, USA) at 25 °C and 100 rpm for 7 days. The grape juice was aeriated for 20 min before fermentation at an airflow of 0.05 mL/min. Overnight non-*Saccharomyces* yeast cultures (6 log CFU/mL) were inoculated into the grape juice and allowed to grow. After 3 days, the commercial *S. cerevisiae* yeast was inoculated (6 log CFU/mL). A pure culture fermentation with the commercial *S. cerevisiae* (Laffort) strain was used as a control. Samples were collected daily for chemical and microbial analysis as described in Section 2.2.2 and Section 2.2.3.

### 2.5. Statistical Analysis

Experiments were carried out in triplicate for two independent assays. Log microbial plate counts and measured concentrations of organic acids, sugar, ethanol, and glycerol were analyzed using the analysis of variance (ANOVA) procedure with Duncan’s multiple-range test with the Statistical Analysis Systems version 9.0 software (SAS Institute, Cary, NC, USA).

## 3. Results and Discussion

### 3.1. Spontaneous Fermentation

The microbiota present in the fresh grape juice and after the 7-day fermentation were characterized. Grape samples were collected in April–May 2013, during the grape harvest in Chile, from Palo Alto Winery (Maule, Chile) and then transported to the experimental laboratory in Santiago (Chile). Upon arrival, grapes were separated from leaves and stems, and processed into juice. The juice was allowed to ferment spontaneously for 7 days.

Fresh grape juice contained 141 ± 0.03 to 138 ± 0.07 g/L glucose, 118.1 ± 0.05 to 120 ± 0.3 g/L fructose, and 0.01 ± 0.00 to 0.03 ± 0.00 g/L glycerol. No ethanol was detected in any of the samples (data not shown). After fermentation (7 days), sugar concentrations were <2 g/L, a value at which the fermented grape juice can be considered as dry and stable to further spoilage. Glycerol concentrations ranged from 10.8 ± 0.03 to 15.7 ± 0.02 g/L, with the higher concentration achieved for the Pinot spontaneous fermentation. Finally, the ethanol concentration ranged from 13.8 ± 0.03 to 14.2 ± 0.01 g/L, with the higher concentration for the Merlot fermentation (data not shown). As expected, LAB and yeast counts increased as the fermentation proceeded (Table 1). LAB final counts after 7 days of fermentation ranged between 6.33 ± 0.01 and 7.70 ± 0.03 log CFU/mL, while yeasts populations reached between 7.39 ± 0.31 and 7.81 ± 0.11 log CFU/mL, depending on the grape cultivar.

### 3.2. Yeasts Diversity during the Spontaneous Fermentation

**Figure 2:**
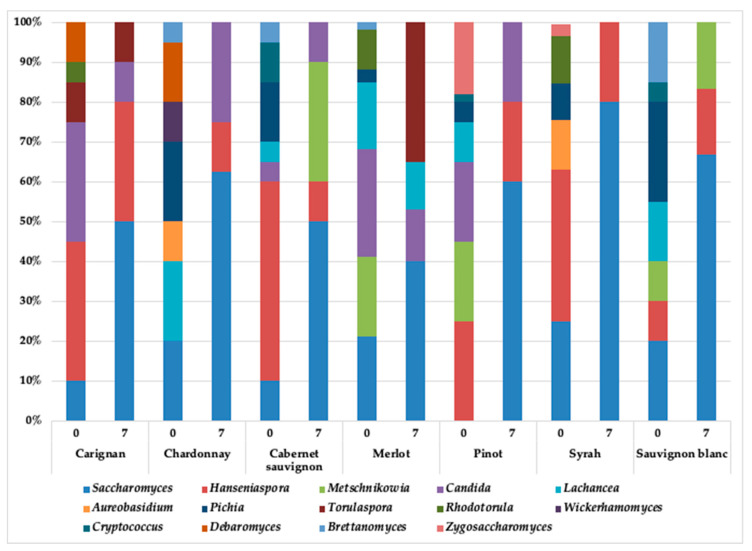
Results of the ITS gene amplicon sequencing data analysis for fresh and fermented grape juice samples collected on days 0 and 7 of fermentation. Data are presented by grape cultivar.

A great diversity of native yeasts was observed (Figure 2). Non-*Saccharomyces* yeasts were abundant in fresh grape juice (day 0 of fermentation), with Pinot, S. Blanc, and C. sauvignon showing the greatest diversity among all grape cultivars. Similarly to our results, Jara et al., (2016) reported several non-*Saccharomyces* yeasts in fresh grape juices from different cultivars in the Maule region (Chile), in which *Metschnikowia*, *Hanseniaspora*, and *Rhodotorula* were the most abundant [51]. Although these genera were also observed in our study, they were not the most abundant in all the grape cultivars we studied, in which a greater diversity was observed.

These difference might be attributed to the different grape cultivars characterized in the studies, while Jara et al., (2016) characterized yeast presence in Petit Verdot, Alicante Bouschet, Torontel, and Mencia cultivars, while we focused on the characterization of Cabernet Sauvignon, Carignan, Chardonnay, Merlot, Pinot, Syrah, and S. Blanc, which present different intrinsic factors, such as nutrient content, that might influence the microbial colonization as well the agronomic practices followed [72,73,74]. In addition, environmental factors, such as rainfall, have been also associated to difference in yeast abundancy [9,11,51]. In grapes, the microbial load is reduced as rainfall increases due to a microorganism wash-out. According to the Environmental Ministry of Chile, no rainfall was reported between March and April 2013, the year during which grape samples were collected for our study. While precipitations were reported for the same months in 2015, during which the study of Jara et al., (2016) took place [75].

The geographical location of the vineyard from which samples were collected might also influence the microbial composition [76]. The Palo Alto vineyard is located in one of the central valleys of Chile (35°26′ S 71°40′ W, Chile, Figure 1), at about 100 m.a.s.l and has a temperature ranging from 17 to 23 °C during the harvest season [77]. Given these conditions, and that the grape vines were collected towards the end of the harvest season, they might have had a greater sugar content, which favors the establishment of a diverse microbial population [78]. In addition, the vinery location is characterized by torrid weather with almost no rainfall from October to March, and rainy winters from April to September. However, the entire region is affected by the “La Niña” climatic event, which operates as an eventual factor that specifically affects the climatic conditions associated with rains causing periodic drought. These conditions not only affect the quality and production of the vineyards, but it is also very possible that they affect the types and proportions of native microorganisms in the grapes [75].

As the spontaneous fermentation progressed, most of the non-*Saccharomyces* yeasts decreased their presence in the must, even disappearing on day 7. This might be explained by its low resistance to the harsh environmental conditions, characterized mostly by the lack of nutrients and high ethanol concentrations [23,24,26,27,49]. On the contrary, the *Saccharomyces* genera that showed a relative abundance from 10% to 40% at day 0 take advantage of these conditions, and therefore become dominant.

In spite of this dominance, some non-*Saccharomyces* yeasts genera were also detected after 7 days of fermentation: *Hansenioaspora*, *Metschnikowia*, *Candida*, *Lachancea,* and *Torulaspora* (Figure 2). The yeast distribution observed was associated with the type of grape juice studied. For example, in the case of the genus *Hanseniaspora,* it was detected in all the grape juices evaluated except in Merlot. *Torulospora* was detected in Carignan and Merlot, while *Metshnikowia* was detected in C. Sauvignon, Sauvignon Blanc, and Pinot. In the case of the *Candida* genus, its presence was found in C. Sauvignon, Merlot, Pinot, and Chardonnay. The presence of these non-*Saccharomyces* genera on day 7 of spontaneous fermentation presupposes the peculiarity of being resistant to ethanol. Although non-*Saccharomyces* yeasts have low tolerance to ethanol, our results show that some can be isolated once the alcoholic fermentation was completed (Table 2). Catrileo, Acuña-Fontecilla, and Godoy (2020) reported that *T. delbrueckii* YCPUC10, isolated from C. Sauvignon grape juice, was able to persist with ethanol levels of about 11% as the result of an adaptative metabolism [79]. Furthermore, certain non-*Saccharomyces* are able to increase their ethanol tolerance by producing succinic acid [80] and this might be the case of the *Metschnikowia, Torulospora, Hanseniospora,* and *Candida* spp. detected at the end of the fermentation. However, more studies are necessary to fully characterize the capability in the isolated strains.

These results are interesting, since they would allow us to investigate local (*terroir*-specific) strains that are able to positively impact the sensory profiles of wines, probably in co-fermentations with *S. cerevisiae,* or even in monocultures. Increases in ethanol resistance might be advantageous in order to allow for the production of other metabolites associated to the non-*Saccaromyces* yeasts. For example, *Metschnikowia* spp. is capable of the formation of esters, monoterpenoids, and higher alcohols due to its β-glucosidase activity, which can improve the aromatic profile in wines [81,82]. *Torulaspora delbrueckii* has been associated to the production of mannoproteins and aroma compounds that increase the mouthfeel in red wines [83,84].

A yeast-like fungus (*Aureobasidium*) was detected with an abundance of approximately 10% in the fresh grape juice samples. Species of *Aureobasidium* have been reported as able to survive in grape juice [23,85] and have been isolated from different grape cultivars in the Maule region [51], so their presence is not unusual. However, in terms of their presence at the end of the fermentation process, this might indicate a contamination problem [23,86].

**Figure 3:**
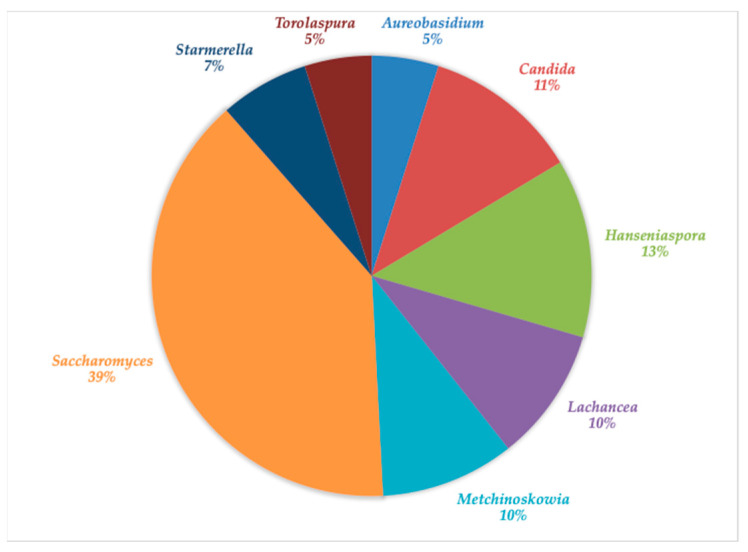
Estimated distribution of yeast species isolated from the spontaneous grape fermentations tested based on morphology. The colonies isolated were identified based on the 26S rRNA sequences.

The culture-dependent analysis showed a diverse make up of yeasts, although less varied than the molecular analysis. Similarly to what was observed with the molecular analysis, the dominant yeast genus in 7-day fermented grape juice was *Saccharomyces* (39%) (Figure 3). However, a significant diversity of non-*Saccharomyces* yeasts was also isolated. The most abundant yeasts were *Hanseniospora* (13%), followed by *Candida* (11%), *Metchinoskowia* (10%), *Lachancea* (10%), *Starmerella* (7%), and *Torolospura* (5%). The yeast-like fungus *Aureobasidium* was also isolated (5%). The yeast-like fungus has also been isolated from spontaneous Grignolino grape fermentation [87]; therefore, their presence is not unusual.

Table 2 shows an abundance of yeasts according to species found in the different musts evaluated; *L. thermotolerans*, *M. pulcherrima*, *H. uvarum*, *T. delbrueckii*, *C. oeophila*, *Starmerella bacillaris* (formerly called C. zemplinina), *C. stellata*, and *A. pulluans*. The Shannon diversity index calculated for each grape cultivar ranged from 0.17 to 1.15 (data not shown), evidencing, as reported previously, the diversity of yeast within different cultivars even if they are cultivated in the same vineyard [87].

Similarly to the molecular analysis, single species distribution was also variable: *T. delbrueckii* was only isolated from C. Sauvignon fermentations; *A. pullulans* from Chardonnay and Syrah; *C. oleophila* from Chardonnay and Merlot; *C. stellata* from Chardonnay, C. Sauvignon, and Merlot; *St. bacillaris* from Carignan, Chardonnay, and Merlot; *M. pulcherrima* from C. Sauvignon, Pinot, and S. blanc; *H. uvarum* from Carignan, C. Sauvignon, Pinot, and Syrah; and *L. thermotolerans* from Carignan, Chardonnay, Merlot, and S. blanc (Table 2). The uneven distribution of yeast species from a single vineyard might be explained by the many microclimates created within the vineyard [11,87]. The non-*Saccharomyes* microbiota was dominated by *L. thermotolerans*, *H. uvarum,* and *M. pulcherrima,* representing 54% of the yeast population. In correlation to the molecular characterization, after 7 days, the Merlot fermentations showed the greatest isolate diversity. On the other hand, as expected, *S. cerevisiae* was isolated transversally in almost all the fermentations, showing the higher numbers of isolates across all grape cultivar samples (Table 2, Figure 3).

The diversity observed in this study is in line with that previously reported for spontaneous grape juice fermentations [10,11,25,26,27,49,87], in which a number of non-*Saccharomyces* yeast were detected and/or isolated primarily at the early stages of the fermentation process. In our study, non-*Saccharomyces* yeasts were also isolated after 7 days of fermentation, which indicates that the species have a certain tolerance to the ethanol concentration (13.08 ± 0.03 to 14.2 ± 0.11% *v*/*v*) and nutrient deprivation (glucose and fructose: 1.70 ± 0.05 to 2.12 ± 0.05 g/L, respectively) conditions reached in 7 days of the spontaneous fermentations (data not shown). Given the scare information available regarding yeast diversity from Chilean grape varieties and *terroirs,* the results obtained contribute to broaden the literature in this topic. Furthermore, with the aim of creating signature and different wines, wineries are exploring the possibility of spontaneous and biodynamic fermentation [88], and therefore, the characterization of the natural microflora and its intrinsic dynamic during the alcoholic fermentation might be crucial to determine correct process that end in stable wines.

### 3.3. Lactic Acid Bacteria Diversity during the Alcoholic Fermentation

**Figure 4:**
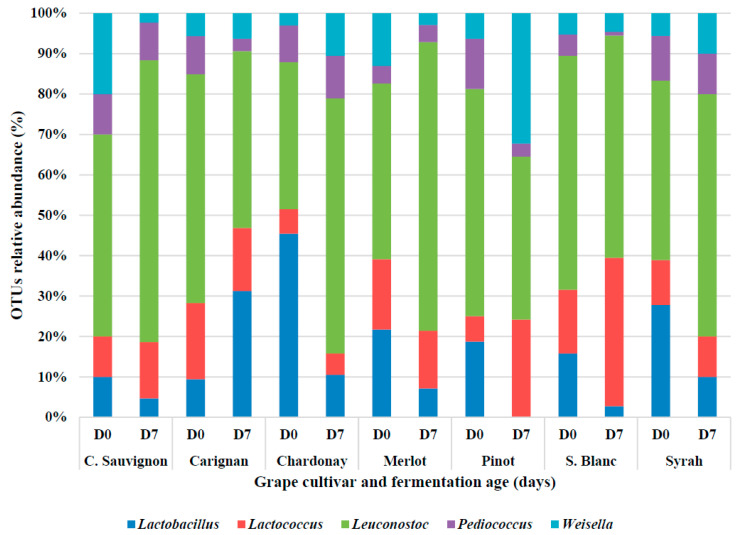
Results of the 16S rRNA gene amplicon sequencing data analysis for the fresh and fermented grape juices collected at days 0 and 7 of the fermentation. Data are presented by grape cultivar.

LAB compose a part of the microbiota found in grapes and winery equipment; they are important in the winemaking process, but their identification and isolation have been limited mostly to grapes and during malolactic fermentation [89]. In our study, the impact of the alcoholic fermentation and the LAB diversity was studied. Five genera were detected in the fresh and fermented grape juices (Figure 4). In fresh grape juice, the dominant genus was *Leuconostoc,* followed by *Lactobacillus*, *Lactococcus*, *Pediococccus,* and *Weisella*. Recently, Kačániová et al., (2020) studied the LAB composition in 10 different grape cultivars (grapes, grape juice, and wine), including Pinot, C. Sauvignon, and Merlot, and reported a similar genera make-up, with *Lactobacillus* as the dominant genera, followed by *Leuconostoc* [90].

In spontaneous fermentations, the bacterial diversity is more abundant than in an inoculated process [48]; however, in our study, the alcoholic fermentation negatively affected the LAB abundance, and after 7 days, *Lactobacillus* and *Pediococcus* decreased significantly (Figure 4). Reduction of LAB populations is expected as the alcoholic fermentation proceeds, the environment becomes richer in ethanol, and sugars are depleted [20]. Although these changes occurred after 7 days of fermentation, *Leuconostoc* remained as the dominant genera. According to Piettet et al., (2011), LAB resistance to ethanol is not strain dependent, and among the 61 LAB tested by the authors, all showed great resistance to ethanol concentrations ranging from 0.5% to 14% *(v*/*v*) [91]. This suggests that an important factor for the reduction in LAB diversity during alcoholic diversity might be the depletion of sugars (20) associated to other factors such as the phenolic content [92]. Similarly to the yeast diversity, the grapes varieties, cultivation conditions, climate, and geographical location of the vineyard affect the LAB composition. For example, Berbegat et al., (2020) reported that the LAB consortium of Uva di Troi grape juices spontaneously fermented, and was composed of *Oenococcus*, *Acetobacter*, *Proponibacterium*, and *Gluconbacter* as the dominant bacteria. *Lactobacillus* was also detected but in lower abundance (10%) [48].

For the culture-dependent analysis, the colonies observed in MRS agar plates were grouped according to their morphology, and these initial observations allowed for the classification of the 58 isolates in three different clusters. Molecular 16S rRNA identification showed that the LAB isolates belonged to the *Leuconostoc* and *Lactobacillus* genera with three species identified (Table 3). The most abundant LAB was *Leuconostoc mesenteroides*, which was isolated from all fresh and fermented grape juice, while *Fructilactobacillus fructivorans* and *Lactobacillus delbrueckii* ssp *delbrueckii* were only isolated from the Carignan and Pinot fresh grape juice, respectively. Berbegal et al., (2019) reported that a heterogenous consortia of LAB are present in spontaneous most fermentations [48]. However, in our study, we were able to observe a lesser diversity, represented mostly by Firmicutes, with the *Leuconostoc* genera predominant. López-Seijas et al., (2020) reported that *Lactobacillus* is the prominent bacteria in Albariño grapes, with *L. plantarum* as the dominant species [93]. In our study, the most abundant species was *L. mesenteroides* isolated at diverse time points in the alcoholic fermentation (Table 3). The lesser diversity might indicate that the spontaneous alcoholic fermentation negatively influences the LAB consortia. This suggests that LAB make-up in the grapes studied shows little tolerance to the conditions reached as ethanol is produced, sugars are depleted, and yeast populations increase. Mendoza, Manc de Nadra, and Farías (2010) reported that, besides the typical conditions achieved during alcoholic fermentation, inhibition might be attributed to the ability of *S. cerevisiae* to produce peptides with inhibitory effects [94]. However, the fact that we were not able to isolate more LAB once the alcoholic fermentation ended, does not indicates that they are not present. According to Capozzi et al., (2021), the concentration of LAB increases 10 to 15 days after the alcoholic fermentation is completed [95]. In our study, we did not consider keeping the fermented grape juice for a MLF fermentation, at which point dormant bacteria might be able to establish. Furthermore, for the culture-dependent method, the medium used to isolate the LAB did not have any enrichment, and the isolation was based in a morphological similarity among the observed colonies, which might also affect the diversity of the isolated species. Thus, more studies are necessary to complete the diversity of LAB during the spontaneous alcoholic and malolactic fermentations of these Chilean grape cultivars.

An important trait of LAB is the production of organic acids such as lactic and acetic acids. Once the fermentation was stopped, lactic acid reached values ranging from 1.12 ± 0.01 to 1.18 ± 0.02 g/L, with the highest concentration associated with the S. Blanc fermentation (Table 4); these values are in line with those previously reported for spontaneous wine fermentations [20]. The higher acetic acid concentration was observed for the Pinot fermentation, which reached 1.17 ± 0.01 g/L, while the rest of the fermentations remained at 1.10 g/L or below. Acetic acid production is expected not only from the native yeasts, but also from the heterofermentative LAB. On the other hand, malic acid decreased in comparison with the initial values, and small amounts were detected once the alcoholic fermentation ended, with values ranging from 0.04 to 0.08 g/L. Ripe grapes might contain between 2 and 6.5 g/L of malic acid; therefore, the presence of small amounts of the acid in the fermented grape juices might be also attributed to the presence of LAB with malolactic activity capacity [20]. Among the isolated species, *Leuconostoc mesenteroides* and *Lactobacillus delbrueckii* have been reported as positive for malolactic activity; therefore, their presence might explain the low malic acid values encountered. The degradation of malic acid in wines results in a decrease in acidity, which favors the overall flavor. In our study, the Carignan and Pinot fermentations resulted in a pH about 0.5 units above the other fermentations (data not shown), which correlates with the presence of these malolactic bacteria (Table 3, Figure 3).

### 3.4. Fermentative Profile of Selected Yeast Isolates

With the aim to better characterize the yeast diversity isolated from the different grape varieties, selected non-*Saccharomyces* yeast isolates were screened for sugar consumption and ethanol yield under limited aerobic conditions over four days of fermentation. Sugar utilization ranged between 14.9% and 63.5% and was strain dependent (Table 5). Ethanol yields, defined as the amount of ethanol produced per gram of consumed sugar, was estimated to identify strains that utilized carbon sources to metabolites different than ethanol. The ethanol yield was isolate specific and ranged from 0.18 to 0.51, while the commercial strain showed a yield of 0.44 (which is expected for the strain). Corresponding to the difference between the control yield, the isolates were grouped as low, medium, and high ethanol producers. Low producers were defined as those isolates that produced equal or less than 0.35 g ethanol/g glucose, medium as those between 0.39 and 0.45 g ethanol/g glucose, and yields higher than 0.45 g ethanol/g glucose were categorized as high producers (Table 5).

One *C. oleophila* strain showed the lowest ethanol yield, interestingly associated to the highest sugar consumption. Non-*Saccharomyces* yeast, in contrast to *S. cerevisiae*, divert the flux of carbon for the production of ethanol to by-products and biomass synthesis [20]. Furthermore, under limited air conditions, a wide array of secondary products are formed [20], which may in part explain the low ethanol yields. One *M. pulcherrima* and three out of five *H. uvarum* strains showed low ethanol yields ranging from 0.30 to 0.35. In Contreras et al., (2015), strains from the same genus showed different ethanol yields, indicating that ethanol production in non-*Saccharomyces* yeasts was strain specific [20]. Some *L. thermotolerans*, *T. delbrueckii*, and *C. oleophila* strains showed ethanol yields similar or higher than *S. cerevisiae,* and therefore were labeled as medium and high ethanol producers, respectively. The first two have been studied as suitable for use in beer and wine fermentation with the trait of reducing the ethanol content [20] and contributing specific volatile profiles [20].

### 3.5. Sequential Fermentations

The yeast species *H. uvarum*, *L. thermotolerans*, *T. delbrueckii*, *C. stellata*, and *M. pulcherrima* have been extensively studied for their use in beer, wine, and the elaboration of other fermented foods [4,20,37,38,41,42,43,47,84,96,97,98,99,100,101], and their beneficial and negative effects in reducing the alcohol content and contributing to the volatile profile and aroma have been reported previously. In contrast, to our knowledge, little information has been reported the *C. oleophila* strain isolated in our study. Therefore, the yeast was selected for further experimentation, along with *C. stellata*, as a yeast control of the same genera yeast.

Several studies have shown that sequential fermentations initiated by non-*Saccharomyces* yeasts, and finished by the inoculation of *S. cerevisiae,* result in wines with desirable and distinct attribute profiles, and with complete fermentations (corroborated by sugar depletion) that result in stable wines [18,41,70,82,98]. With this in mind, *C. olephila*, and *C. stellata* were fermented in sequence with the commercial *S. cerevisiae* in Carménère grape juice. The grape variety was selected because it is the signature grape in the Chilean wine industry. The variety disappeared from Europe during the middle of the 19th century and reappeared years later in Chile [71]. The Chilean vineyards in which it is produced have a particular climate and *terroir* conditions that enable the production of the variety. Moreover, there is an increasing interest of the regional wine industry to give additional attributes to these signature wines, and the use of non-conventional yeast might aid in this purpose.

**Figure 5:**
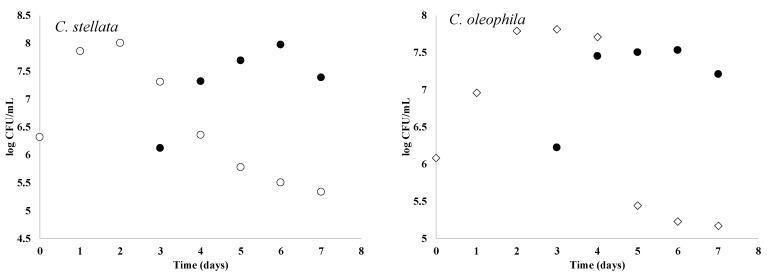
Cell counts in sequential fermentations. ● *S. cerevisiae*; ◦ *C. stellata*; ◊ *C. oleophila*. Values represent mean ± standard deviations for two independent sets and three replicates (*n* = 6). Dotted line at day 3 indicates the time point at which *S. cerevisiae* was inoculated.

Figure 5 shows the growth curves obtained for the different isolates and the *S. cerevisiae* strain. As expected, after inoculation, the non-*Saccharomyces* isolates increased in population, reaching a peak after 2 days; thereafter, the yeast populations decreased significantly to finish at day 7 of fermentation with values below 5.5 log CFU/mL for all the strains (Figure 4). The performance observed for the *Candida* spp. studied here was similar to those reported when native yeasts from spontaneous Pinot Noir, Coralin, Chardonnay, Resi, Petit Arvine, Etmitage, and Gudel fermentations were studied [20]. In contrast, *S. cerevisiae*, inoculated at day 3, increased its population for 3 days straight and maintained concentrations above 7 log CFU/mL until the end of the fermentation. 

The sequential fermentations resulted in lower ethanol concentrations compared to the control fermentation (pure *S. cerevisiae* inoculation) (Table 6). The average ethanol concentrations were 11.8% and 12.5% for *C. stellata* and *C. oleophila*, respectively (Table 6). Before the *S. cerevisiae* inoculation, ethanol content in the *C. stellata* fermentations reached 7.70 ± 0.05% (*v*/*v*), and 4.55 ± 0.08% (*v*/*v*) for *C. olephila*, which correlates with the low ethanol-producing capacity showed by these strains in the defined medium (Table 5). Decreases in the non-*Saccharomyces* yeast populations were observed once the *S. cerevisae* inoculum increased in population. These is expected since *S. cerevisiae* is highly effective in ethanol production, and therefore, the ethanol concentrations in the fermented grape juice increase. Domizzo et al., (2007) reported that among the non-*Saccahromyces* yeasts characterized in their study, the *Candida* spp. showed the greatest population reduction once *S. cerevisiae* was inoculated [102].

The addition of the *S. cerevisiae* allowed the wine to complete the sugar utilization, and therefore increased the ethanol content, but concentrations were below 14% (*v*/*v*). Since Chilean wines are characterized by their strong ethanol content with 14% (*v*/*v*) as the average concentration, the use of the selected strains in sequential fermentation with *S. cerevisiae* might be an alternative for the production of reduced alcohol wines. Reduction of alcohol is of interest given that high concentrations of ethanol mask some wine attributes [70]. In addition, economical and health trends have influenced this market, and nowadays there a few vineyards that produce lower alcohol content wines.

Acetic acid production was similar for *C. stellata* and *C. oleophila,* with concentrations of 0.52 and 0.53 g/L, respectively (Table 7). The ability to produce acetic acid from sugar consumption has been reported before for *Candida* spp.; however, the values reported here are higher to those reported previously. Díaz et al., (2013) reported acetic acid concentrations ranging from 0.024 to 0.27 g/L. Although the acetic acid production might be considered a sign of microbial spoilage, *C. stellata* have not been reported as spoilage-causing agents. Moreover, the presence of the yeast that are able to persist towards the end of the alcoholic fermentations is associated with the enhancement of the sensory profile of the wine [103]. Compared to the control fermentations the sequential procedure resulted in higher acetic, malic, and succinic acid concentrations, which is consistent with what was previously reported in the literature [38,41,46,104].

Succinic acid concentrations (Table 7) were detected in the range of 1.99 to 2.39 g/L. Although the concentrations fall above the usual range in wine (0.5 to 1.5 g/L), for some red wine varieties, concentrations higher that 3 g/L have been reported without exerting a negative effect in the wine flavor [41,70,105].

Malic acid production was strain specific with 0.08 ± 0.13, 0.06 ± 0.05, and 0.05 ± 0.05 g/L for *C. stellata* and *C. oleophila*, respectively, and all values were lower than the control fermentation (Table 7). These values are much lower than those reported for other non-*Saccharomyces* cultivated in limited aeration systems [70,104]. Some non-*Saccharomyces* yeasts have been reported as able to control the acidity of wines by metabolizing organic acids [106,107]. However, to our knowledge, no information for malic acid utilization or production for *C. stellata* and *C. oleophila* in wines has been reported before. However, the values reported here might indicate that the use of the yeast strains could result in less acidic wines, and even more, might help to reduce or eliminate the malolactic fermentation needed for the wines’ stability. However, more studies are necessary to confirm this.

In the sequential fermentations *C. oleophila* produced the highest glycerol content (Table 7), approximately two times higher than the other two sequential fermentations. Glycerol is a non-volatile compound that is formed by sugar consumption during the yeast metabolism. The compound contributes to wine with sweetness and fullness by increasing the density and viscosity of the wine. The glycerol values achieved in the sequential fermentations are higher to those reported for other non-*Saccharomyces* yeasts in single, sequential, and co-inoculated fermentations [20]. The redirecting of ethanol production to other metabolites, including glycerol, has been reported as an approach to reduce the ethanol concentration. The selected yeast strains used in this study showed ethanol yields of 0.18 and 0.40 for *C. oleophila* and *C. stellata*, respectively (Table 5), and therefore, the increased glycerol concentration might be attributed to the yeast´s ability to canalize sugar consumption into glycerol production. *C. stellata* fermentative potential has been studied not only in wines, but also in beer and vinegar production [21,26,108]. The fructophilic yeast metabolism is characterized by the production of glycerol instead of ethanol, and thus produces wines with reduced alcohol content [20]. On the other hand, to our knowledge, the use of *C. oleophila* for wine production has not been previously reported. Our results suggests that the yeasts have a potential for the production of reduced alcohol wines in sequential fermentations; however, more studies are necessary to determine the effect on aroma and sensory characteristics of the resulting wines.

## 4. Conclusions

The seven grape cultivars (C. Sauvignon, Carignan, Chardonnay, Merlot, Pinot, S. Blanc, and Syrah) studied showed a great native yeast diversity in the fresh grape juice. Although *S. cerevisiae* became the dominant yeast after 7 days of fermentation, some non-*Saccharomyces* yeast were able to persist the ethanol conditions and were isolated from the fermented grape juices. In order of prevalence, species from *Metschnikowia*, *Lachancea*, *Hanseniaspora*, *Candida*, and *Toulaspora* were detected and isolated. The yeast-like fungus *A. pullulans* was also isolated, although its presence was encountered in low abundance. Three LAB species were also isolated. Two of them (*Le. mesenteroides* and *L. delbrueckii*) have been previously reported as malolactic positive, and might be responsible, along with the yeast population, for the low malic acid concentration achieved during the alcoholic fermentation, but more studies to confirm this ability, and its performance in co-culture or sequential fermentations with yeasts are needed.

The selected non-*Saccharomyces* yeasts showed a wide range of ethanol yields that were strain dependent. Among the isolated yeast, two *Candida* species were further studied to characterize the yeasts’ ability to ferment in sequential fermentations with *S. cerevisiae*. The fermentations with the *Candida* species resulted in Carménère fermented grape juices with reduced ethanol concentrations (10–12% (*v*/*v*)), in comparison with the commercial *S. cerevisiae* strain, which presented ethanol content close to 14% (*v*/*v)*. Regardless of the ethanol content, less acetic and malic concentrations were observed when compared to the control fermentations, which might positively impact the final wine acidity. High glycerol production in the fermentation with *C. oleophila* was observed, which might directly impact the wine mouthfeel. To our knowledge, this is the first time *C. oleophina* has been used for grape juice fermentations, and given its fermentation profile, the yeast might be a suitable candidate for wine production; however, the impact on wine flavor and the potential of producing desired (or not) aroma and flavor compounds need to be further studied.

## Figures and Tables

**Table 1:** Changes in lactic acid bacteria and yeast population observed during the spontaneous grape juices fermentation.

Grape Variety	LAB (log CFU/mL)		Yeast (log CFU/mL)	
	D0	D7	D0	D7
C. Sauvignon	2.13 ± 0.06	7.80 ± 0.07	4.81 ± 0.07	7.81 ± 0.11
Carignan	2.32 ± 0.05	6.33 ± 0.01	4.69 ± 0.08	7.52 ± 0.23
Chardonnay	2.38 ± 0.08	7.77 ± 0.16	5.08 ± 0.00	7.71 ± 0.05
Merlot	1.81 ± 0.90	7.50 ± 0.06	5.19 ± 0.02	7.71 ± 0.05
Pinot	1.69 ± 0.08	7.59 ± 0.30	4.63 ± 0.03	7.39 ± 0.31
S. Blanc	1.72 ± 0.11	7.35 ± 0.03	4.75 ± 0.12	7.67 ± 0.11
Syrah	1.82 ± 0.17	7.70 ± 0.03	4.89 ± 0.14	7.60 ± 0.12

Values represent mean ± standard deviations for two independent sets and three replicates (*n* = 6).

**Table 2:** Identification of the yeast species found on day 7 of spontaneous fermentation. These species were obtained from isolated colonies that presented a different and unique morphology, which was later identified with 26S rRNA sequences.

Grape Variety	Yeast Identification (% Abundance)
Carignan	*Hanseniaspora uvarum (40)*
	*Saccharomyces cerevisiae (20)*
	*Starmerella bacillaris (20)*
	*Lachancea thermotolerans (20)*
Chardonnay	*Aureobasidium pullulans (16)*
	*Candida oleophila (12)*
	*Candida stellate (12)*
	*Starmerella bacillaris (12)*
	*Hanseniospora uvarum (12)*
	*Lachancea thermotolerans (12)*
	*Saccharomyces cerevisiae (24)*
Cabernet Sauvignon	*Candida stellata (24)*
	*Hanseniaspora uvarum (20)*
	*Torolaspura delbrueckii (7)*
	*Metchinoskowia pulcherrima (21)*
	*Saccharmyces cerevisiae (28)*
Merlot	*Candida stellata (15)*
	*Starmerella bacillaris (15)*
	*Candida oleophila (15)*
	*Lachancea thermotolerans (25)*
	*Saccharomyces cervisiae (15)*
	*Torulaspora delbrueckii (15)*
Pinot	*Hanseniospora uvarum (20)*
	*Metschnikowia pulecherrima (20)*
	*Saccharomyces cerevisiae (60)*
Syrah	*Aureobasidium pullulans (17)*
	*Hanseniospora uvarum (17)*
	*Saccharomyces cerevisiae (66)*
Sauvignon blanc	*Lachancea thermotolerans (14)*
	*Metschnikowia pulcherrima (28)*
	*Saccharomyces cerevisiae (58)*

**Table 3:** Identification of the LAB colonies isolated from the spontaneous grape juice fermentations. Colonies piqued represented a unique colony morphology. The colonies isolated were identified based on the 16S rRNA sequences.

Grape Variety (No. of Colonies)	Isolation Time Point (Day)	LAB Identification
C. Sauvignon (6)	0, 7	*Leuconostoc mesenteroides*
Carignan (4)	0, 7	
Chardonnay (3)	0, 7	
Merlot (6)	0, 7	
Pinot (11)	0, 3, 7	
S. Blanc (17)	0, 3, 7	
Syrah (3)	0, 7	
Carignan (3)	0	*Fructilactobacillus fructivorans*
Pinot (5)	0	*Lactobacillus delbrueckii ssp delbrueckii*

**Table 4:** Organic acids produced after 7 days of spontaneous fermentation.

Grape	Lactic Acid (g/L)		Acetic Acid (g/L)		Malic Acid (g/L)	
	D0	D7	D0	D7	D0	D7
C. Sauvignon	ND	1.15 ± 0.01 ^a^	ND	1.07 ± 0.03 ^a^	2.50 ± 0.01 ^a^	0.04 ± 0.01 ^a^
Carignan	ND	1.18 ± 0.04 ^a^	0.03 ± 0.01	1.10 ± 0.05 ^b^	2.61 ± 0.01 ^b^	0.04 ± 0.01 ^c^
Chardonnay	ND	1.12 ± 0.01 ^a^	ND	1.06 ± 0.02 ^a^	2.32 ± 0.01 ^c^	0.08 ± 0.02 ^b^
Merlot	ND	1.14 ± 0.03 ^a^	ND	1.05 ± 0.01 ^a^	3.13 ± 0.01 ^e^	0.06 ± 0.02 ^d^
Pinot	0.08 ± 0.03 ^a^	1.17 ± 0.04 ^b^	ND	1.17 ± 0.04 ^b^	2.37 ± 0.03 ^c^	0.06 ± 0.01 ^c^
S. Blanc	0.03 ± 0.02 ^b^	1.13 ± 0.02 ^a^	ND	1.05 ± 0.03 ^a^	2.85 ± 0.01 ^d^	0.07 ± 0.02 ^b^
Syrah	ND	1.12 ± 0.05 ^a^	ND	1.06 ± 0.03 ^a^	2.82 ± 0.01 ^d^	0.08 ± 0.01 ^b^

Values represent mean ± standard deviations for two independent sets and three replicates (*n* = 6). Different lower-case superscript letters within columns indicate significant differences (*p* < 0.05).

**Table 5:** Ethanol yield and sugar utilization of non-*Saccharomyces* yeast isolates.

Yeast Isolate	Grape Source	Ethanol Yield (g Ethanol/g Glucose)	% Glucose Consumed	Category
*Candida olepohila*–1	Chardonnay	0.18	63.50%	LOW
*Candida oleophila*–2	Merlot	0.23	50.90%	
*Hanseniaspora uvarum*–1	Carignan	0.3	23.40%	
*Metschnikowia pulcherrima*–1	C. Sauvignon	0.31	22.70%	
*Hanseniaspora uvarum*–2	Chardonnay	0.33	20.50%	
*Hanseniaspora uvarum*–3	C. Sauvignon	0.33	20.50%	
*Metschnikowia pulcherrima*–2	Pinot	0.33	21.20%	
*Metschnikowia pulcherrima*–3	Pinot	0.33	37.60%	
*Hanseniaspora uvarum*–4	Pinot	0.34	21.60%	
*Metschnikowia pulcherrima*–4	S. blanc	0.35	42.70%	
*Metschnikowia pulcherrima*–5	S. blanc	0.35	41.60%	
*Starmerella bacillaris*	Carignan	0.39	35.00%	MEDIUM
*Candida stellata*	Chardonnay	0.4	42.00%	
*Hanseniaspora uvarum*–5	Syrah	0.4	23.70%	
*Torulaspora delbrueckii*–1	C. Sauvignon	0.41	41.90%	
*Lanchacea thermotolerans*–2	Merlot	0.43	33.70%	
*Saccharomyces cerevisiae*	Control	0.44	98.50%	
*Aureobasidium pullulans*–1	Syrah	0.45	26.40%	
*Candida oleophila*–3	Chardonnay	0.45	20.00%	
*Lanchacea thermotolerants*–2	Carignan	0.45	32.70%	
*Torulaspora delbrueckii*–2	Merlot	0.45	30.70%	
*Aureobasidium pullulans*–2	Chardonnay	0.47	27.00%	HIGH
*Hanseniaspora uvarum*–6	C. Sauvignon	0.48	14.90%	
*Lanchacea thermotolerans*–3	Carignan	0.48	31.70%	
*Lanchacea thermotolerans*–4	Merlot	0.48	29.70%	
*Aureobasidium pullulans*–3	Chardonnay	0.51	30.00%	
*Lanchacea thermotolerans*–5	S. blanc	0.51	35.30%	

**Table 6:** Sugar utilization and ethanol production observed during the sequential fermentation of *C. stellata* and *C. oleophila* with *S. cerevisiae*.

Yeast	Glucose (g/L)			Fructose (g/L)			Ethanol % (*v/v*)		
	Fermentation Day *								
	0	3	14	0	3	14	0	3	14
*C. stellata*	139.2 ± 0.08 ^a^	49.2 ± 0.01 ^a^	1.90 ± 0.05 ^a^	110.1 ± 0.01 ^a^	50.0 ± 0.01 ^a^	1.00 ± 0.03 ^a^	ND	7.70 ± 0.05 ^a^	10.8 ± 0.1 ^a^
*C. oleophila*	140.1 ± 0.03 ^a^	453.1 ± 0.12 ^b^	1.12 ± 0.01 ^b^	110.0 ± 0.02 ^a^	58.2 ± 0.08 ^b^	0.85 ± 0.07 ^a^	ND	4.55 ± 0.08 ^b^	12.5 ± 0.1 ^b^
*S. cerevisiae*	139.5 ± 0.03 ^a^	5.13 ± 0.03 ^b^	ND	109.4 ± 0.01 ^a^	15.31 ± 0.02 ^d^	1.46 ± 0.09 ^c^	ND	12.8 ± 0.03 ^d^	13.5 ± 0.67 ^c^

Values represent mean ± standard deviations for two independent sets and three replicates (*n* = 6). Different lower-case superscript letters within columns indicate significant differences (*p* < 0.05). * Non-*Saccharomyces* yeasts were single inoculated at time point 0, and after 3 days, *S. cerevisiae* was inoculated into the fermentation flasks.

**Table 7:** Organic acids and glycerol observed in the *C. stellata* and *C. oleophila*, and sequential fermentations with *S. cerevisiae*.

Yeast	Acetic Acid	Malic Acid	Succinic Acid	Glycerol
	(g/L)			
*C. stellata*	0.52 ± 0.07 ^a^	0.82 ± 0.13 ^a^	1.99 ± 0.15 ^a^	10.3 ± 0.05 ^a^
*C. oleophila*	0.53 ± 0.11 ^a^	0.65 ± 0.15 ^b^	2.31 ± 0.01 ^b^	18.1 ± 0.05 ^b^
*S. cerevisiae*	0.26 ± 0.01 ^c^	1.03 ± 0.03 ^d^	1.21 ± 0.05 ^d^	10.4 ± 2.04 ^a^

Values represent mean ± standard deviations for two independent sets and three replicates (*n* = 6) collected after 7 days of fermentation. Different lower-case letters within columns indicate significant differences (*p* < 0.05).

## Data Availability

No public data was used.
